# Central Nervous System and Limb Embolism Concurrence due to Atrial Myxoma: A Case Report‏

**Published:** 2017-07

**Authors:** Javad Salimi, Khosro Najari, Pezhman Farshidmehr, Roja Toosi, Batool Naghavi, Tayeb Ramim

**Affiliations:** 1 *Sina Hospital, Tehran University of Medical Sciences, Tehran, Iran.*; 2 *Tehran University of Medical Sciences, Tehran, Iran.*; 3 *Cancer Pharmacogenetics Research Group (CPGRG), Iran University of Medical Sciences, Tehran, Iran.*

**Keywords:** *Heart neoplasms*, *Myxoma*, *Lower extremity*, *Ischemia*, *Stroke*, *Embolism*

## Abstract

Cardiac myxomas are the most common cardiac tumors with diverse nonspecific clinical manifestations. A 78-year-old man presented to the emergency department with complaints of pain and coldness of the left lower extremity. The left femoral artery pulse was detected, while the pulses of the left popliteal, dorsalis pedis, and posterior tibialis arteries were absent. No blood inflow was detected in the superficial and deep femoral, popliteal, and anterior and posterior tibialis arteries. Thrombectomy was performed, and a fatty-like mass from the bifurcation of the common femoral artery and a thrombotic mass from the proximal portion of the superficial and deep femoral arteries were removed. The pulsatile inflow and palpable pulses of the left femoral, popliteal, dorsalis pedis, and posterior tibialis arteries were restored after surgery. The histological findings of the embolus were suggestive of a cardiac myxoma. The patient’s consciousness and lower limb blood flow improved gradually. He was discharged from the hospital with full awareness and improved lower extremity muscle function 2 weeks after surgery.

## Introduction

Cardiac myxomas are uncommon heart tumors originating from the endocardium. Between 60% and 80% of cardiac myxomas are diagnosed in the left atrium.^1^ The average age of the clinical manifestations of cardiac myxomas has been reported to be between 30 and 70 years old. The clinical presentations of these tumors include obstructive cardiac signs, embolic signs, and constitutional presentations. Moreover, 50% of the embolic manifestations tend to present with neurological signs as a result of cerebral ischemia.^2^ Cardiac myxomas are the most common primary cardiac tumors in adults. These tumors are benign and slow-growing neoplasms which usually do not metastasize and infiltrate into the myocardium.^3^ The incidence of these tumors is estimated at approximately 0.5-1 cases per 1,000,000 individuals per year, with a female-to-male ratio of 3 to 1.^4^ The majority of cardiac myxomas are diagnosed between 4 and 6 decades of life.^5^ Between 60% and 80% of cardiac myxomas are diagnosed in the left atrium,^1^ 15-28% in the right atrium, 8% in the right ventricle, and 3-4% in the left ventricle.^6^

We describe a patient who had a sudden onset of left lower extremity ischemia and stroke and was found to have a left atrial cardiac myxoma.

## Case Report

A 78-year-old man presented to the emergency department of Sina Hospital, Tehran, Iran, with complaints of pain and coldness of the left lower extremity, which had suddenly occurred at 11 a.m. On arrival at our emergency department at 2 p.m., he was conscious. Bradycardia (heart rate = 45/min) and high blood pressure (165/80 mmHg) were detected. The respiratory rate and temperature were normal. On examination, the left lower extremity was cold. The left femoral artery pulse was detected, but the pulses of the left popliteal, dorsalis pedis, and posterior tibialis arteries were absent. Impaired sensory examination was noted. The muscle force of the left lower limb was 4/5. At baseline, laboratory tests revealed hemoglobin of 13.4 mg/dL, platelet of 294,000 cells/µL, white blood cell of 8,100 cells/µL, urea of 24 mg/dL, and serum creatinine of 1.06 mg/dL. Arterial blood gas showed no significant abnormal findings. Electrocardiography revealed sinus bradycardia. On color Doppler sonography of the left limb, the common femoral artery had high resistance and stenotic blood inflow as far as 2 centimeters above the bifurcation of the common femoral artery. No blood inflow was detected in the superficial and deep femoral, popliteal artery, and anterior and posterior tibialis arteries. A mildly hyperechoic thrombosis was seen in the superficial and deep femoral arteries. The findings were suggestive of acute arterial obstruction.

**Figure 1:**
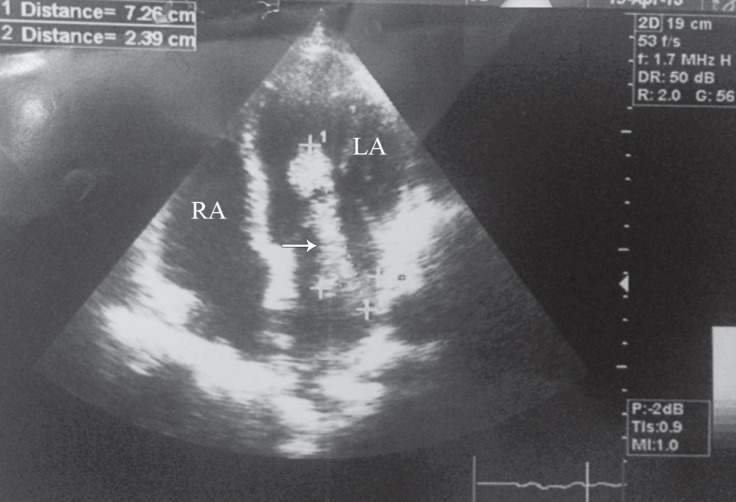
Transthoracic echocardiogram reveals the presence of a large mobile mass in the left atrium protruding into the left ventricle (arrow).

RA, Right atrium; LA, Left atrium

**Figure 2:**
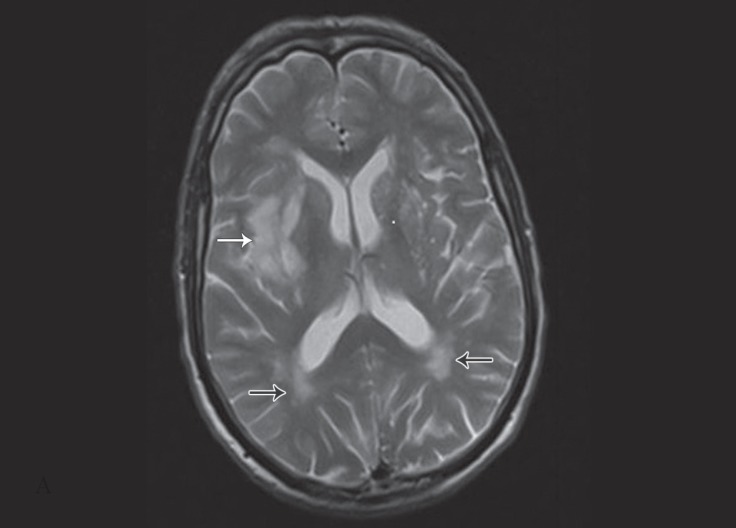
Brain magnetic resonance imaging without contrast shows an abnormal bright signal in the periventricular white matter in both cerebral hemispheres, in favor of microvascular ischemic changes (black arrows). Also, acute infarction can be seen in the right insula, external capsule, putamen, and caudate nucleuses (arrow).

While the patient was under observation in the emergency department, he had a sudden onset of loss of consciousness and left hemiplegia. On examination, the pupils were normal in size and reactive to light. Aphasia and facial asymmetry were noted. The upper and lower left limbs were plegic. After neurology and cardiovascular consultation, echocardiography and brain computed tomography (CT) scan were done. Echocardiography revealed a large mobile mass in the left atrium protruding into the left ventricle (Figure 1). Brain CT scan was negative for neurological findings; therefore, brain magnetic resonance imaging (MRI) without contrast was requested. On MRI, an abnormal bright signal in the periventricular white matter in both cerebral hemispheres in favor of microvascular ischemic changes was reported. In addition, acute infarction was detected in the right insula, external capsule, putamen, and caudate nucleuses (Figure 2). After primary treatment, angiography was performed; it demonstrated a normal inflow in the right and left iliac arteries and the aorta. A cutoff was shown in the proximal portion of the common femoral artery, and runoff was reported in the deep femoral artery and the proximal portion of the superficial arteries. Subsequently, the patient underwent emergent surgery.

**Figure 3:**
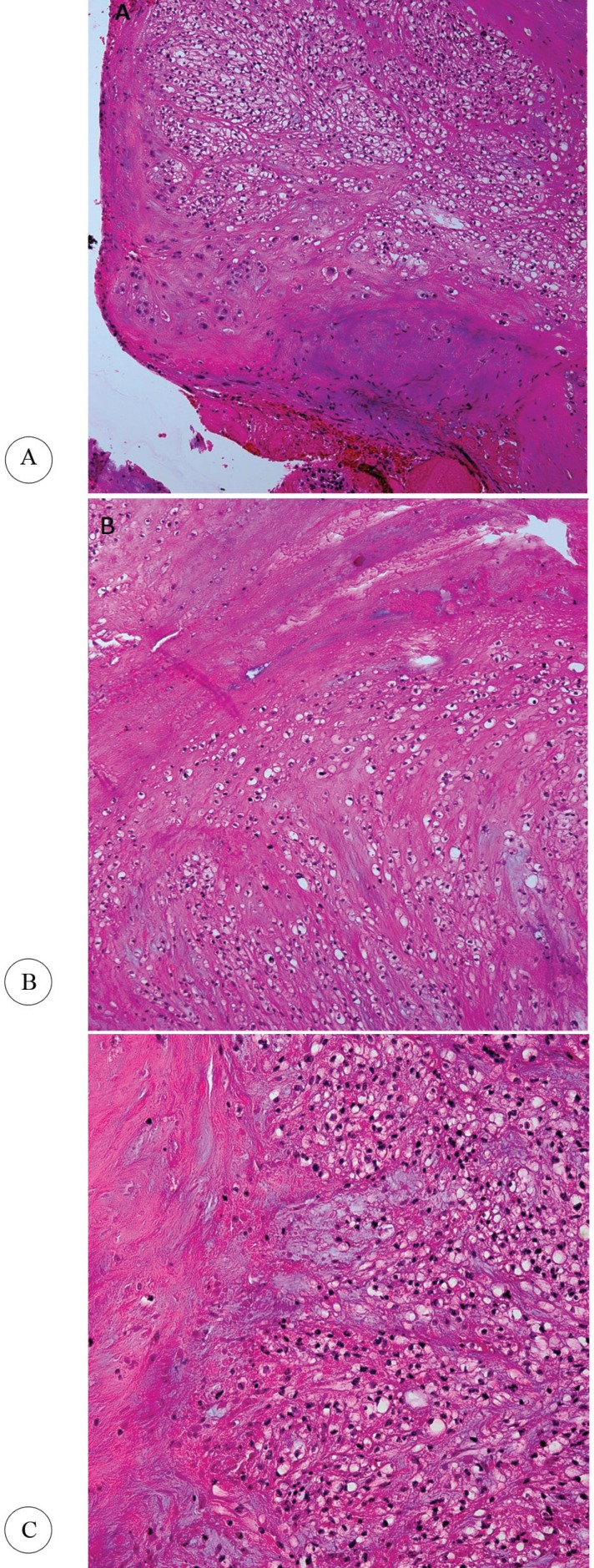
Histopathological features of the embolus removed from the bifurcation of the common femoral artery ([A & B] × 100, and [C] × 200) are consistent with cardiac myxoma.

After local anesthesia, an incision was made over the left inguinal area. Thrombectomy was performed with a Fogarty catheter (3, 4 French). A fatty-like mass from the bifurcation of the common femoral artery and a thrombotic mass from the proximal portion of the superficial and deep femoral arteries were removed. The pulsatile inflow and palpable pulses of the left femoral, popliteal, dorsalis pedis, and posterior tibialis arteries were restored after surgery. The specimen was sent to the pathology center of Sina Hospital. The histological findings of the embolus were suggestive of a cardiac myxoma (Figure 3). The patient was referred to the cardiac surgery department for further evaluation and treatment. After open-heart surgery, the patient was hospitalized for 10 days in the intensive care unit. Gradually, the patient’s consciousness and lower limb blood flow improved. He was discharged from the hospital with full awareness and improved lower extremity muscle function 2 weeks after surgery.

## Discussion

The clinical manifestations of cardiac myxomas depend on their location, size, mobility, and overall morphology.^7^ Some patients have no symptoms, especially when the tumor size is small; the tumor is diagnosed incidentally in these patients.^8^ Cardiac myxomas usually present with one or more of the following manifestations: hemodynamic derangement after an intracardiac obstruction, symptoms of systemic embolisms (peripheral and cerebral) or pulmonary embolization, and constitutional symptoms such as weakness, fatigue, anorexia, lethargy, loss of appetite, loss of weight, and low-grade fever (in 90% of the cases).^8^ Fever of unknown origin has been reported as a rare presentation of cardiac myxomas.^9^

Embolic manifestations occur in 30-50% of patients with cardiac myxomas. Due to the higher prevalence of the left-sided location of cardiac myxomas, systemic embolism (peripheral and cerebral) often occurs in the retinal and cerebral arteries as well as in lower limb, renal, visceral, and coronary arteries, and sometimes even in the abdominal aorta.^10^ Lower extremities are the second anatomical site to become involved in the left-sided location of cardiac myxomas as a result of embolism.^11^ There are some reported cases of sudden-onset lower limb ischemia and abdominal pain due to the occlusion of the aorta.^12^^-^^14^

More than 50% of embolic events due to cardiac myxomas involve the central nervous system and retinal arteries, leading to the obstruction of the intracranial and extracranial vessels as well as visual impairment, cerebral infarction, seizure, cerebral necrosis, intracranial aneurysms, hemiparesis, aphasia, and progressive dementia.^15^ Loss of consciousness and stroke as a result of the carotid artery occlusion has also been reported.^2^

Cardiac myxomas are usually diagnosed via echocardiography. It is recommended that cardiac myxomas be surgically resected soon as possible in order to prevent embolism.^2^ Acute lower limb ischemia and stroke are rare simultaneous presentations of cardiac myxomas. Our patient was a 78-year-old man presenting first with acute left lower limb ischemia findings, consistent with the obstruction of the bifurcation of the left common femoral artery. During close observation and treatment, the patient experienced sudden-onset loss of consciousness and left hemiplegia. At baseline, brain CT scan showed no abnormal findings. Further evaluation revealed ischemia in the periventricular white matter in the cerebral hemispheres, right insula, external capsule, putamen, and caudate nucleuses on MRI. Echocardiography showed a large mobile mass in the left atrium protruding into the left ventricle. Pathological examination of the specimen from embolectomy confirmed cardiac myxoma.

## Conclusion

The presence of a cardiac myxoma should be suspected in patients with the acute onset of arterial occlusion signs and symptoms such as pain, paresthesia, poikilothermia, and paralysis. In addition, stroke should be considered as a common manifestation of cardiac myxomas. Early diagnosis of cardiac myxomas may dramatically diminish the morbidity and mortality. Surgery should be performed as soon as possible to remove emboli.

## Notes:

***This paper should be cited as:*** Salimi J, Najari K, Farshidmehr P, Toosi R, Naghavi B, Ramim T. Central Nervous System and Limb Embolism Concurrence due to Atrial Myxoma: A Case Report‏. J Teh Univ Heart Ctr 2017;12(3):145-148.
